# Microstructure and Hydrothermal Stability of Microporous Niobia-Silica Membranes: Effect of Niobium Doping Contents

**DOI:** 10.3390/membranes12050527

**Published:** 2022-05-17

**Authors:** Jiachen Xia, Jing Yang, Hao Zhang, Yingming Guo, Ruifeng Zhang

**Affiliations:** 1 School of Urban Planning and Municipal Engineering, Xi’an Polytechnic University, Xi’an 710048, China; xiajiachen02@163.com (J.X.); guoyingming@xpu.edu.cn (Y.G.); ruifengzhangtry@xpu.edu.cn (R.Z.); 2 Xi’an Thermal Power Research Institute Co., Ltd., Xi’an 710032, China; zhanghao@mail.tpri.com.cn

**Keywords:** niobium doping, calcination temperature, Nb/SiO_2_ membrane, H_2_ permselectivities, hydrothermal stability

## Abstract

Methyl-modified niobium-doped silica (Nb/SiO_2_) materials with various Nb/Si molar ratios (n_Nb_) were fabricated using tetraethoxysilane and methyltriethoxysilane as the silica source and niobium pentachloride as the niobium source by the sol–gel method, and the Nb/SiO_2_ membranes were prepared thereof by the dip-coating process under an N_2_ calcining atmosphere. Their microstructures were characterized and gas permeances tested. The results showed that the niobium element existed in the formation of the Nb-O groups in the Nb/SiO_2_ materials. When the niobium doping content and the calcining temperature were large enough, the Nb_2_O_5_ crystals could be formed in the SiO_2_ frameworks. With the increase of n_Nb_ and calcination temperature, the formed particle sizes increased. The doping of Nb could enhance the H_2_/CO_2_ and H_2_/N_2_ permselectivities of SiO_2_ membranes. When n_Nb_ was equal to 0.08, the Nb/SiO_2_ membrane achieved a maximal H_2_ permeance of 4.83 × 10^−6^ mol·m^−2^·Pa^−1^·s^−1^ and H_2_/CO_2_ permselectivity of 15.49 at 200 °C and 0.1 MPa, which also exhibited great hydrothermal stability and thermal reproducibility.

## 1. Introduction

Environmental protection and resource shortage are nowadays issues that need to be faced in the process of world development. The continuous growth of the world’s population has led to the increasing consumption of the earth’s resources. Some experts have suggested hydrogen as an alternative fuel because of its zero pollution. Nowadays, hydrogen is primarily produced from fossil fuels, such as natural gas and coal through steam reforming/gasification and water gas shift reactions [1]. In order to obtain high purity hydrogen from either syngas or the products of the water-gas shift reaction, separation of H_2_ from other gases such as CO_2_, CO, or CH_4_ is necessary. Consequently, hydrogen purification from the above CO_2_-containing reaction gas mixture is becoming an important issue. After long-term research and efforts by scientists, a large number of experimental results have shown that the membrane separation technology has shown great potential in gas separation. An inorganic membrane has the advantages of good resistance to high temperature and pressure, high mechanical strength, good chemical stability, long service life, and resistance to halite, which make it attractive in the field of gas separation [2]. In the last two decades, H_2_-separation membranes have been developed using various materials, such as palladium and its alloys, silica, alumina, etc. In the research to date, inorganic silica membranes, especially those derived from the sol-gel technique, are some of the best among the various inorganic materials for the separation of hydrogen-containing gas mixtures.

However, in high temperature and humid air, pure SiO_2_ membranes showed poor hydrothermal stability. The Si-O-Si bonds were broken and Si-OH bonds were formed when inter-played with water, resulting in the densification of the silica structure [3,4]. Many scientists have carried out a lot of research work to improve the hydrothermal stability of silica membrane materials. In recent years, the two primary methods have been the introduction of groups such as F^–^, Cl^–^, -C_n_H_2n_, -C_n_H_2n+1_, phenyl groups, etc. [5,6], and the doping of transition metals such as nickel [7,8,9], palladium [10,11], zirconium [12], niobium [13], magnesium [14], aluminum [15], cobalt [16,17], etc. Wei et al. [6] prepared a perfluorodecyl-modified silica membrane by the sol-gel method using tetraethylorthosilicate (TEOS) and 1H, 1H, 2H, and 2H-perflouorodecyltriethoxysilane (PFDTES) as precursors. The H_2_ permeance of the as-prepared membrane was 9.71 × 10^−7^ mol·m^−2^·s^−1^·Pa^−1^, and the H_2_/CO_2_ permselectivity and binary gas separation factor were 7.19 and 12.11, respectively. Under humid conditions with a temperature of 250 °C and a water vapor molar ratio of 5%, the single H_2_ permeance and H_2_/CO_2_ permselectivity remained almost constant for at least 200 h.

Among the transition metals, niobium doping has caught researchers’ eyes. Boffa et al. [18] prepared niobia-silica membranes using tetraethyl orthosilicate (TEOS) as the Si source and niobium penta (n-butoxide) as the Nb source. Their research results showed that the hydrothermal stability of the microporous niobia-silica membranes was better than that of the pure SiO_2_ membrane because the incorporation of Nb ions into the silica matrix. Qi et al. [13] prepared a novel microporous hybrid silica membrane using 1,2-bis (triethoxysilyl) ethane (BTESE) and niobium penta (n-butoxide) as precursors for the permselectivity of CO_2_. The result showed that the permselectivity of H_2_/CO_2_ for the Nb-BTESE membrane could be tuned by altering the calcination temperature. Lin et al. [19] investigated the influence of the sol particle size on the gas permselectivity of the niobium-doped hybrid silica membrane. The prepared Nb/SiO_2_ membrane had an H_2_ permeability of 8.36 × 10^−8^ mol·m^−2^·s^−1^·Pa^−1^ with a mean particle size of 5 nm. However, there have been few studies focusing on the effects of niobium doping content on the microstructures and gas permeances of niobium-doped SiO_2_ membranes.

In this work, we prepared a kind of new niobium-doped SiO_2_ membrane using tetraethoxysilane (TEOS) and methyltriethoxysilane (MTES) as the Si sources and niobium pentachloride (NbCl_5_) as the niobium source. Nb/SiO_2_ membranes with different Nb/Si molar ratios (n_Nb_) were prepared. The effects of n_Nb_ and calcination temperature on the microstructures of Nb/SiO_2_ materials were studied in detail. Characterization and results were attained by Fourier transform infrared (FTIR) spectroscopy, X-ray diffraction (XRD), X-ray photo electron spectroscopy (XPS), N_2_ adsorption/desorption measurements, and scanning electron microscopy (SEM). The gas permeation tests and hydrothermal stability of the Nb/SiO_2_ membranes were performed and are discussed.

## 2. Experimental

### 2.1. Fabrication of Nb/SiO_2_ Sols

The Nb/SiO_2_ sols were prepared using tetraethoxysilane (TEOS) and methyltriethoxysilane (MTES) as the silica sources, niobium pentachloride (NbCl_5_) as the niobium source, absolute ethanol (EtOH) as the solvent, and HCl as the catalyst. High purity solid NbCl_5_ powder was dissolved in absolute ethanol to obtain a 0.43 M NbCl_5_ solution. This process was carried out in a fume hood, stirring and dissolving with a glass rod until the HCl gas was released. The reaction equation is as follows:

NbCl_5_ (s) + nC_2_H_5_OH (l) → NbCl_5-n_(OC_2_H_5_)_n_ (l) + nHCl (g)

According to the mol ratio of TEOS:MTES:EtOH:H_2_O:HCl:NbCl_5_ = 1:0.8:16:7:0.085:n_Nb_, the EtOH, TEOS, and MTES were first mixed with the solution of NbCl_5_ solution in an ice bath and stirred magnetically for 40 min. After that, a mixture of HCl and water was carefully added drop-wise and then refluxed in a water bath at 60 °C for 3 h. In this way, the Nb-doped SiO_2_ sols were obtained. The n_Nb_ is the molar ratio of Nb/TEOS, which was 0, 0.08, 0.2, and 1, respectively. The Nb-doped SiO_2_ sol with n_Nb_ = 0 is also referred to as the SiO_2_ sol. The Nb-doped SiO_2_ sols were diluted three times using absolute ethanol to obtain the final Nb/SiO_2_ sols.

### 2.2. Fabrication of Nb/SiO_2_ Materials

The as-prepared Nb/SiO_2_ sols were dried in a vacuum oven to prepare the dry gels. The obtained dry gels were then ground into fine powders and calcined under N_2_ atmosphere in a temperature-controlled tubular furnace at various temperatures (200 °C, 400 °C, 600 °C, 800 °C) for 2 h with a ramping rate of 0.5 °C·min^−1^. The final niobium-doped silica (Nd/SiO_2_) materials were produced. The Nb/SiO_2_ materials with n_Nb_ = 0 were also referred to as the SiO_2_ materials.

### 2.3. Fabrication of Nb/SiO_2_ Membranes

**Figure 1:**
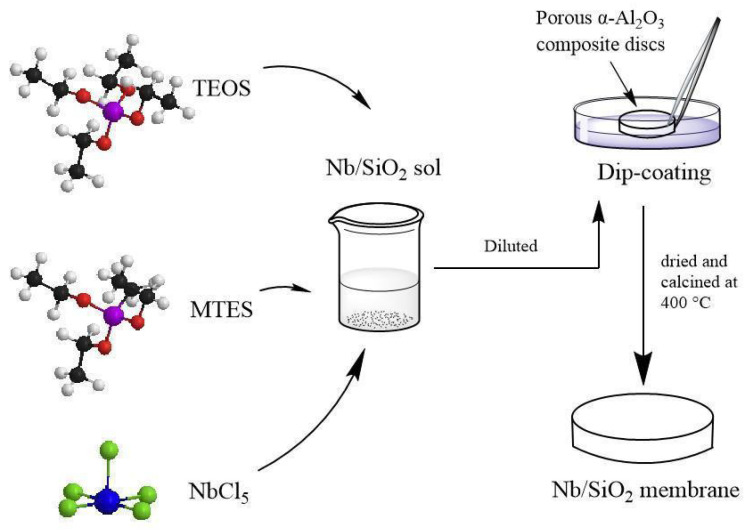
Schematic diagram of the fabrication of Nb/SiO_2_ membranes by the sol–gel process.

To obtain the Nb/SiO_2_ membranes, part of the above Nb/SiO_2_ sols were applied to the surface of porous α-Al_2_O_3_ composite discs (Hefei Shijie Membrane Engineering Co., Ltd., Hefei, China) by the dip-coating method. The discs each had a thickness of 4 mm, a diameter of 30 mm, a mean pore diameter of 100 nm, and a porosity of 40%. The dipping time was 6 s. Four-layer Nb/SiO_2_ membranes were prepared, and each Nb/SiO_2_ layer was individually calcined under N_2_ atmospheres in a temperature-controlled furnace to 400 °C at a ramping rate of 0.5 °C·min^−1^ and with a dwell time of 2 h. The prepared Nb/SiO_2_ membranes were used to test the permeances of H_2_, CO_2_, and N_2_; the preparation process is shown in Figure 1.

### 2.4. Steam-Treatment and Regeneration of Nb/SiO_2_ Membranes

The steam stability of the membranes was tested by exposure to saturated steam at 25 °C for 10 d. The thermal regeneration of Nb/SiO_2_ membranes after steam treatment was carried out at a calcination temperature of 350 °C by the same calcining procedure as described above. After the steam-treatment and regeneration, the gas permeances of Nb/SiO_2_ membranes were tested, respectively.

### 2.5. Characterization

The sol densities were determined using a pycnometer. The solid contents of sols were determined by the weighing method. The particle size distributions of Nb/SiO_2_ sols were measured using a Malvern Nano ZS size analysis instrument (Malvern Instruments Ltd., Malvern, UK) The functional groups of samples were characterized by Fourier transform infrared spectroscopy (FTIR, Nicolet 5700, Thermo Nicolet Corporation, Fitchburg, WI, USA), and the wavelength measurement range was 400–4000 cm^−1^ as determined by the KBr compression method. The material phase structure was determined by a Rigaku D/max-2550 pc X-ray diffractometer (XRD, Rigaku D/max-2550, Hitachi, Tokyo, Japan) with CuKα radiation under the conditions of 40 kV and 40 mA. The chemical components of Nb/SiO_2_ samples were performed by an X-ray photoelectron spectrometer (XPS, ESCALAB250xi, Thermo Scientific, Waltham, MA, USA) with AlKα excitation. The morphologies of surfaces and cross-sections for the membranes were observed by scanning electron microscopy (SEM, JEOL JSM-6300, Hitachi, Tokyo, Japan) under 5 kV acceleration voltage. Before the SEM tests, the samples were treated with gold spraying. The BET surface area and pore volume of the samples were measured by N_2_ sorption/desorption isotherms with a specific surface area and pore and pore-size analyzer (ASAP 2020, Micromeritics, Norcross, GA, USA).

**Figure 2:**
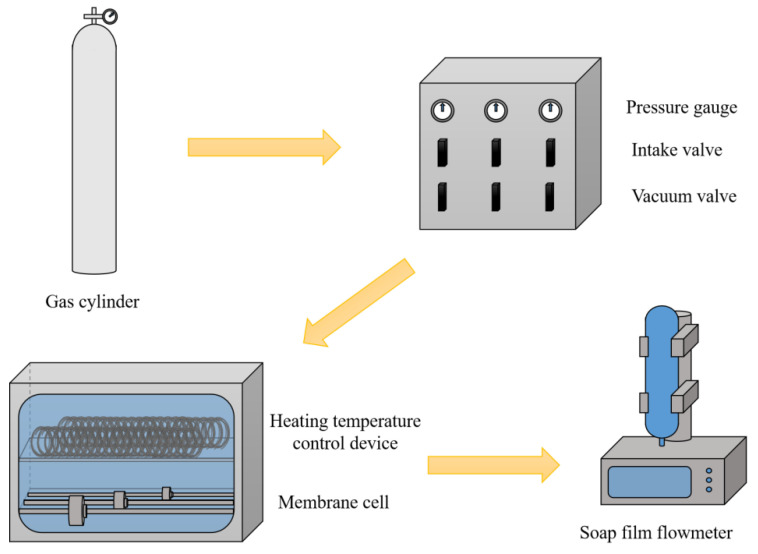
Schematic diagram of the experimental setup.

Single gas permeances of Nb/SiO_2_ membranes were measured under a transmembrane pressure of 0.1-0.4 MPa using the schematic diagram of the experimental setup shown in Figure 2. Prior to the gas permeation measurement, the membranes were mounted in a stainless-steel module with a cylindrical geometry and placed inside the furnace in the gas permeation measurement rig at a temperature range of 25 to 200 °C. The membranes were tested using single gases with different kinetic diameters (H_2_, CO_2_, and N_2_), and permeance was measured by using a soap film flow meter. The gas permselectivities, also known as ideal selectivities, were calculated by the permeance ratio between two gases. The steam stability of the membranes was tested by exposure to saturated steam at 25 °C for 10 d. The thermal regeneration of Nb/SiO_2_ membranes after steam stability testing was carried out at a calcination temperature of 350 °C by the same calcining procedure as described above. It should also be noted that permeate gas flow was only recorded after a steady state was attained.

## 3. Results and Discussion

### 3.1. Analysis of Nb/SiO_2_ Sol Performance

**Figure 3:**
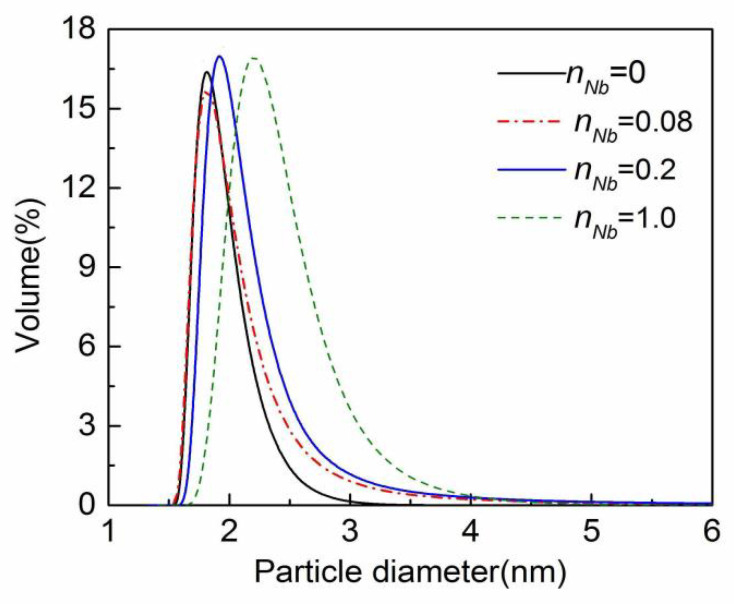
The particle size distributions of Nb/SiO_2_ sols with various n_Nb_.

The influence of n_Nb_ on the pH value, density, and solid content of Nb/SiO_2_ sol is shown in Table 1. The particle size distributions of Nb/SiO_2_ sols with various n_Nb_ are depicted in Figure 3. As seen in Table 1, with increasing n_Nb_, the sol pH value decreased while the density and solid content increased. As seen in Figure 3, with the increase of n_Nb_, the mean particle sizes of Nb/SiO_2_ sols increased, and their particle size distributions became wider. The particle size distributions of Nb/SiO_2_ sols with n_Nb_ = 0, 0.08, and 0.2 were narrow, and their mean particle sizes were small. However, when n_Nb_ was increased to 1.0, the mean particle size of Nb/SiO_2_ sol increased greatly. In the experiment, the prepared NbCl_5_ solution was acidic. The increase of n_Nb_ in Nb/SiO_2_ sol made the acidity of the sol increase, which sped up the hydrolysis–polycondensation reaction. As a result, the sol density, solid content, and particle size increased. On the other hand, with the occurrence of the hydrolysis-polycondensation reaction, Nb-O-Si bonds were formed gradually. The radius of the niobium atom (2.08 Å) is larger than that of the Si atom (1.46 Å), and the bond length of Nb-O is longer than that of Si-O (the bond lengths of Nb-O and Si-O are about 2.13 and 1.64 Å, respectively), which contributed to the increase of sol particles.

### 3.2. Chemical Structure Analysis

**Figure 4:**
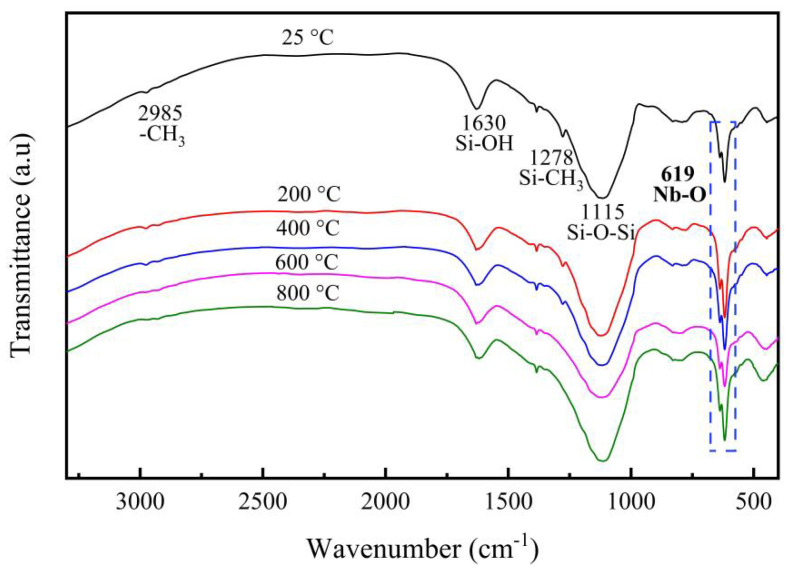
FTIR spectra of Nb/SiO_2_ materials with n_Nb_ = 1 calcined at various temperatures.

The phase-chemical structure of Nb/SiO_2_ materials may be influenced by the introduced Nb element, so that the effect may have been more evident in the samples containing higher Nb contents. In order to study the effect of calcination temperature on Nb/SiO_2_ materials, the Nb/SiO_2_ samples with n_Nb_ = 1 were used for the measurement analysis. Figure 4 shows the FTIR spectra of Nb/SiO_2_ materials with n_Nb_ = 1 at different calcination temperatures. In Figure 4, the absorption peaks located at 1055 and 788 cm^−1^ were associated with the Si-O-Si bonds. The peaks at 2985 cm^−1^ and 1630 cm^−1^ were assigned to the mode of -CH_3_ groups and Si-OH bonds, respectively [11]. The band at 1278 cm^−1^ was designated as Si-CH_3_ groups. The intensity of the absorption peaks at 2985 cm^−1^ and 1278 cm^−1^ decreased obviously with increases in the calcination temperatures and disappeared as the calcination temperature reached 600 °C, indicating that the -CH_3_ groups had been broken down at this temperature. The absorption peak observed at 619 cm^−1^ corresponded to the Nb-O bonds, which enhanced in intensity with the increase of calcination temperatures.

**Figure 5:**
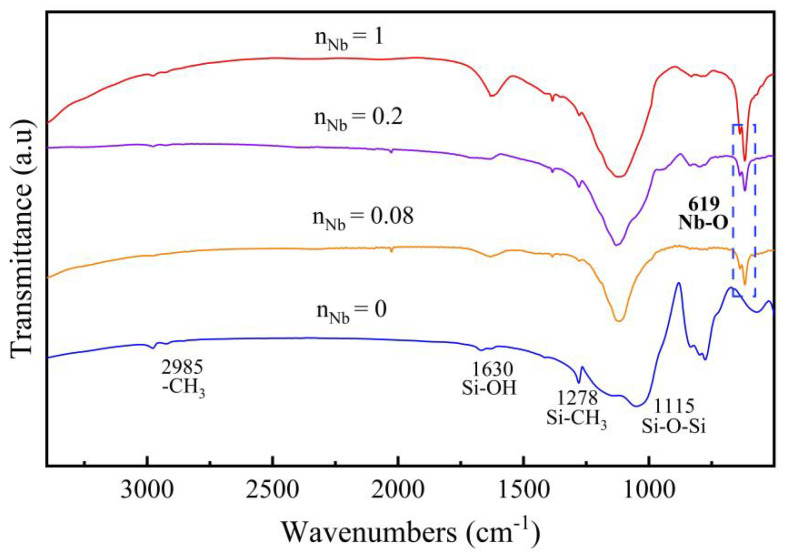
FTIR spectra of Nb/SiO_2_ materials with various n_Nb_ calcined at 400 °C.

The FTIR spectra of Nb/SiO_2_ materials with various n_Nb_ calcined at 400 °C are provided in Figure 5. It can be seen from Figure 5 that the hydrophobic Si-CH_3_ groups located at 1278 cm^−1^ were all involved in the Nb/SiO_2_ materials with various n_Nb_. The absorption peak locations of Nb/SiO_2_ materials with n_Nb_ = 0.08, 0.2, and 1 were roughly the same, but the intensities of individual peaks were different. The peak located at 619 cm^−1^ corresponding to the Nb-O groups did not exist in the pure SiO_2_ materials. This certified that the Nb had been successfully incorporated into the silica frameworks. At the same time, the peak intensity due to the Nb-O groups became strong continuously with the increase of niobium doping.

### 3.3. Phase Structure Analysis

**Figure 6:**
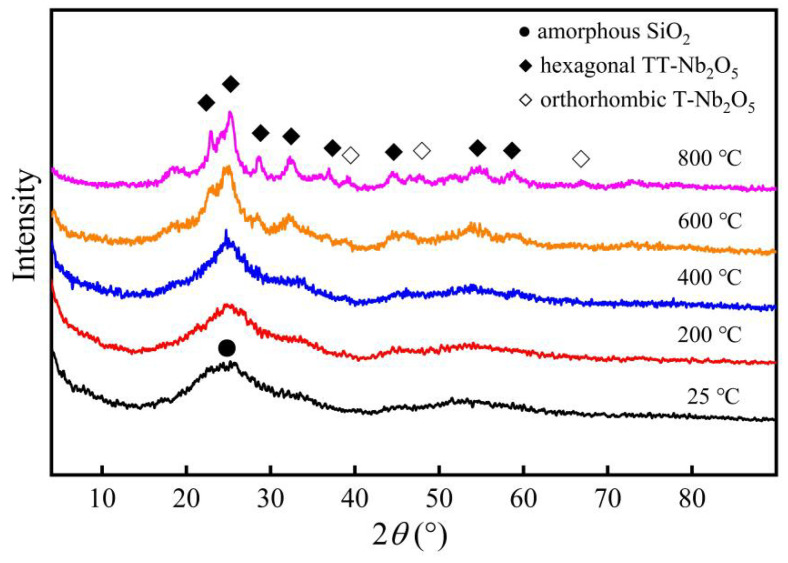
XRD patterns of Nb/SiO_2_ materials with n_Nb_ = 1 calcination at various temperatures.

The XRD patterns of Nb/SiO_2_ materials with n_Nb_ = 1 calcined at various temperatures are given in Figure 6. The broad diffraction peak at the range of about 2*θ* = 20–30° was assigned to the amorphous SiO_2_. An obvious crystallization peak of Nb_2_O_5_ was detected when the calcination temperature was increased to 600 °C. According to the conclusion from Kosutova, there will be two new crystalline phases formed sequentially when the sample is heated up to 450 °C [20]. The first crystalline phase was identified as hexagonal TT-Nb_2_O_5_ (JCPDS No-400-028-0317) [21]. The TT notation comes from the German Tief-Tief (low–low), referring to the temperature at which the structure was observed in the sequence of niobium oxides obtained at elevated temperatures, first used in Ref [22]. In Figure 6, the diffraction peaks at 2*θ* = 22.6°, 28.6°, 36.8°, 46.2°, 50.8°, and 55.2° were assigned to the (001), (180), (201), (002), (380), and (182) crystal planes, respectively, of hexagonal TT-Nb_2_O_5_, which indicated the formation of hexagonal TT-Nb_2_O_5_ (JCPDS No.00-030-0873). In Figure 6, another phase transition was observed at 800 °C, which was associated with the transition from hexagonal TT-Nb_2_O_5_ to orthorhombic T-Nb_2_O_5_ (JCPDS No. 404-007-0752) [23]. The transformation caused the splitting of the diffraction peaks [24]. It is evident that a high calcining temperature can improve the crystallinity of Nb_2_O_5_ species.

**Figure 7:**
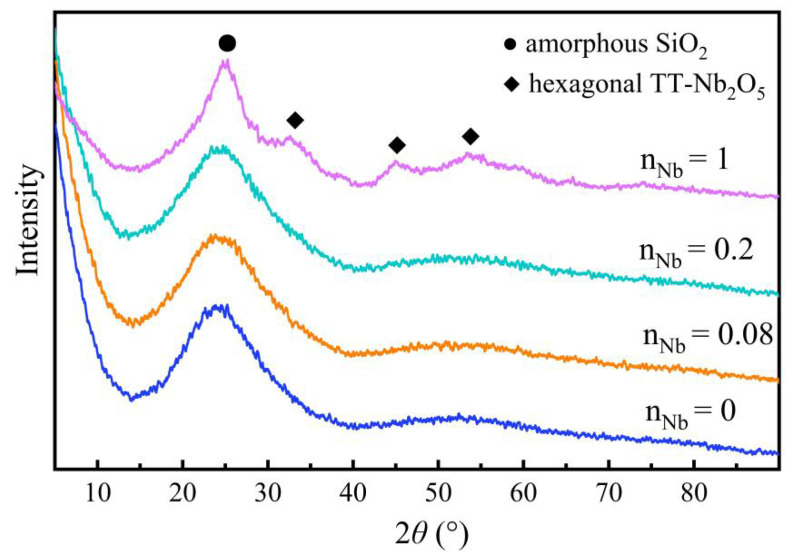
XRD patterns of Nb/SiO_2_ materials doped with various n_Nb_ calcined at 400 °C.

The XRD patterns of the Nb/SiO_2_ materials with various n_Nb_ calcined at 400 °C are provided in Figure 7. From Figure 7, it can be seen that as the n_Nb_ ≤ 0.2, the XRD curves of the Nb/SiO_2_ materials were similar, and only the diffraction peak between 20° and 30° corresponding to the amorphous SiO_2_ could be observed. When the n_Nb_ was increased to 1, a new diffraction peak assigned to the hexagonal TT-Nb_2_O_5_ appeared besides for the amorphous SiO_2_. This means there was no crystalline form of hexagonal TT-Nb_2_O_5_ when the Nb-doped content was low. When the niobium doping content and the calcining temperature were large enough, the Nb_2_O_5_ crystals could be formed in the SiO_2_ frameworks.

**Figure 8:**
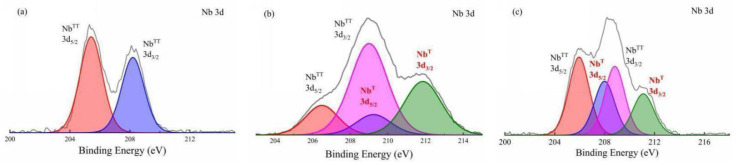
The Nb 3d XPS patterns of Nb/SiO_2_ materials with n_Nb_ = 1 calcined at various temperatures: (**a**) 400 °C, (**b**) 600 °C, and (**c**) 800 °C.

In order to explore the crystal form of niobium, the Nb/SiO_2_ materials with n_Nb_ = 1 calcined at various temperatures were characterized by XPS, which are shown in Figure 8. In Figure 8, the Nb^TT^ 3d and Nb^T^ 3d mean the Nb-O of the hexagonal crystal system TT-Nb_2_O_5_ and orthorhombic crystal system T-Nb_2_O_5_, respectively. It can be seen that the Nb^TT^ 3d_5/2_ peak and the Nb^TT^ 3d_3/2_ peak appeared at 206.4 eV and 209.1 eV, respectively, in the sample calcined at 400 °C. The data matched with the species of niobium oxide, which proved the formation of Nb_2_O_5_ crystals. The temperature related to the formation of the TT-Nb_2_O_5_ phase was reported to be approximately 500 °C or higher, according to the studies conducted on nanostructures or thin films [24,25]. Furthermore, the calcination temperature reached 600 °C, and the Nb^T^ 3d_5/2_ and Nb^T^ 3d_3/2_ peaks also appeared at 209.0 eV and 211.8 eV, respectively, which were the Nb 3d peaks of orthorhombic phase T-Nb_2_O_5_. The conclusion was as same as that of XRD analysis.

### 3.4. SEM Analysis

**Figure 9:**
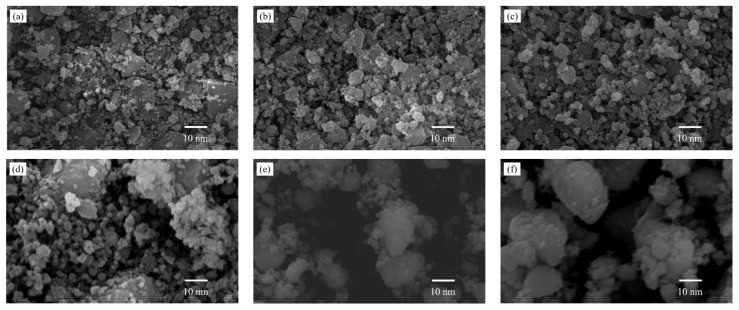
SEM image of Nb/SiO_2_ materials with various n_Nb_ calcinations at various temperatures: (**a**) n_Nb_ = 0, 400 °C; (**b**) n_Nb_ = 0.08, 400 °C; (**c**) n_Nb_ = 0.2, 400 °C; (**d**) n_Nb_ = 1, 400 °C; (**e**) n_Nb_ = 1, 600 °C; (**f**) n_Nb_ = 1, 800 °C.

Figure 9 shows the SEM images of Nb/SiO_2_ materials with various n_Nb_ calcined at various temperatures. From Figure 9a–d, it can be seen that when calcined at 400 °C, the samples had the morphology of nanoparticles with dispersed amorphous structures, and the particle sizes increased with increasing n_Nb_.

Comparing Figure 9d with Figure 9e,f, it can be clearly observed that the morphology and particle size of Nb/SiO_2_ material with n_Nb_ = 1 underwent a large change after being calcined at 600 °C, and the materials had a tendency to develop from an amorphous state to a spherical shape. It can be seen from Figure 9f that when the calcination temperature reached 800 °C, the materials exhibited a spherical structure, the particle size increased, and there were more small-sized spherical particles formed around the large particles. In summary, with the increase of calcination temperature and Nb-doping, the particle sizes increased.

**Figure 10:**
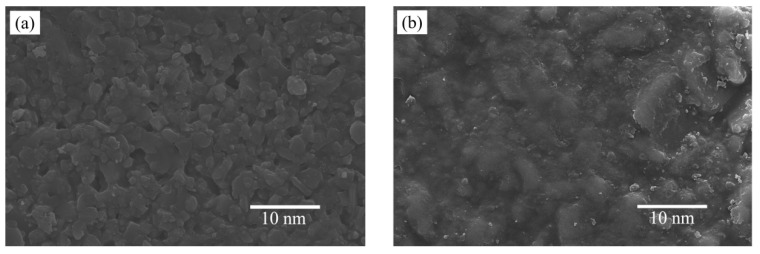
SEM image of Nb/SiO_2_ membranes with n_Nb_ = (**a**) 0 and (**b**) 0.08 calcined at 400 °C.

Moreover, the SEM images of the pure SiO_2_ membrane and Nb/SiO_2_ with n_Nb_ = 0.08 calcined at 400 °C are depicted in Figure 10. From Figure 10 it can be observed that there were no visible cracks or pinholes on the membrane surfaces, indicating that the membranes were well coated. It could be seen that niobium doping could make particle sizes increase.

### 3.5. Pore Structure Analysis

**Figure 11:**
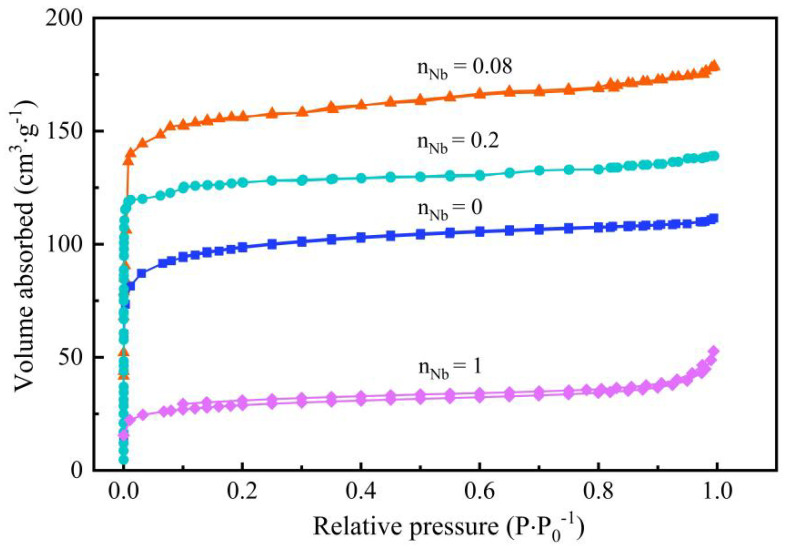
N_2_ adsorption–desorption isotherms of Nb/SiO_2_ materials doped with various n_Nb_ calcined at 400 °C.

The pore properties of the as-prepared Nb/SiO_2_ materials were investigated by N_2_ adsorption/desorption to characterize the surface area, pore volume, and porosity. Figure 11 shows the N_2_ adsorption/desorption isotherms of Nb/SiO_2_ materials with various n_Nb_ calcined at 400 °C under an N_2_ atmosphere. After calcining, all isotherms displayed similar tendencies, which were similar to a tape I isotherm. It could be proved that the Nb/SiO_2_ materials all exhibited the adsorption isotherm characteristics of microporous materials. This material was subjected to a strong force due to the gas in the micropores, so it could quickly reach the absorption saturation state.

**Figure 12:**
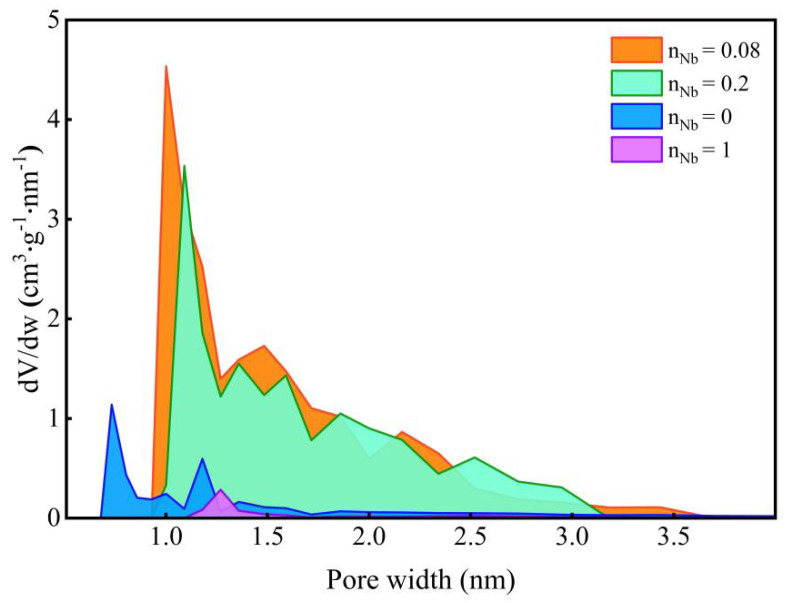
Pore size distribution of Nb/SiO_2_ materials doped with various n_Nb_ calcined at 400 °C.

Figure 12 manifests the pore size distribution of Nb/SiO_2_ materials with various n_Nb_ calcined at 400 °C. As shown in Figure 12, as n_Nb_ ≤ 0.2, with the Nb doping, the pore size of Nb/SiO_2_ materials were widened, and the microporous structure was maintained. When n_Nb_ = 1, the Nb/SiO_2_ material became dense.

The pore structure parameters of Nb/SiO_2_ materials with various n_Nb_ calcined at 400 °C are shown in Table 2. It can be observed that with the increases of n_Nb_, the mean pore size, BET surface area, and total pore volume increased until n_Nb_ = 0.08, and then they began to decrease. This is because the bond length of Nb-O is longer than that of Si-O (the bond lengths of Nb-O and Si-O are about 2.13 and 1.64 Å, respectively). The formation of the Nb-O bond helped the formation of larger particles. Thus, with the increase of the Nb doping amount, the particle size increased, and the formed pore grew gradually. However, when n_Nb_ was >0.08, that changed. Some small hexagonal TT-Nb_2_O_5_ particles were formed and distributed in the SiO_2_ network, which led to a decrease in the mean pore size, BET surface area, and total pore volume

### 3.6. Gas Permeation and Separation Property Analysis

Based on all the results of the above, the Nb-doping content showed a significant impact on the microstructures of Nb/SiO_2_ materials. Compared with the Nb/SiO_2_ membrane with n_Nb_ = 0.08 and n_Nb_ = 0.2, the pore volume of the Nb/SiO_2_ membrane with n_Nb_ = 1 was too small, which is bad for gas separation. Therefore, the Nb/SiO_2_ membrane with n_Nb_ = 1 was not considered here.

**Figure 13:**
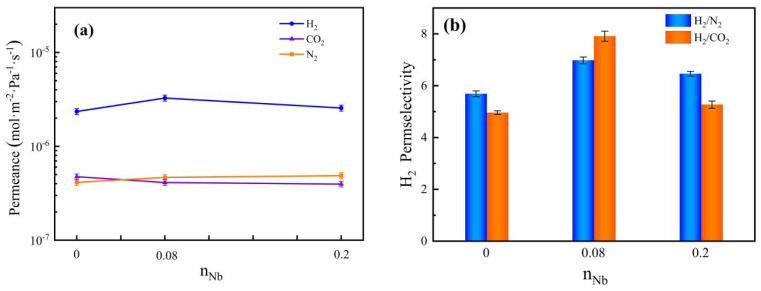
Effect of n_Nb_ on (**a**) gas permeance and (**b**) H_2_ permselectivity of Nb/SiO_2_ membrane at a pressure difference of 0.1 MPa and 25 °C.

A transient test was conducted on the Nb/SiO_2_ membranes with various n_Nb_ at 25 °C and a differential pressure of around 0.1 MPa, and the gas permeances and H_2_ permselectivities are shown in Figure 13. In Figure 13a, the H_2_ and N_2_ permeances of Nb/SiO_2_ membranes with n_Nb_ = 0.08 and 0.2 were greater than those of the pure SiO_2_ membrane, while the change trends of CO_2_ permeances were the contrary. Compared with the pure SiO_2_ membrane, the H_2_ permeances of Nb/SiO_2_ membranes with n_Nb_ = 0.08 and 0.2 increased by 39.0% and 8.9%, respectively. In Figure 13b, the permselectivities of H_2_/CO_2_ and H_2_/N_2_ for the pure SiO_2_ membrane were greater than the values based on Knudsen diffusion (4.69 and 3.74, respectively), which means the transport in pure SiO_2_ membrane is controlled by molecular sieving plus Knudsen diffusion. The H_2_/CO_2_ and H_2_/N_2_ permselectivities for the Nb/SiO_2_ membranes with n_Nb_ = 0.08 and 0.2, respectively, were obviously higher than those of the pure SiO_2_ membrane. Compared with the pure SiO_2_ membrane, the H_2_/CO_2_ permselectivity for the Nb/SiO_2_ membranes with n_Nb_ = 0.08 and 0.2 increased by 59.5% and 6.3%, respectively. The larger mean pore size and higher pore volume of Nb/SiO_2_ membranes with n_Nb_ = 0.08 and 0.2 than those of the SiO_2_ membrane can explain the increase of gas permeance. The larger mean pore size of Nb/SiO_2_ membrane indicated that the increase of H_2_/CO_2_ permselectivity was not due to molecular sieving. This suggests that the doping of Nb introduced another transport mechanism. Some researchers have proposed that doping transition metals into the microporous SiO_2_ network will generate Lewis acids on the surface of the membrane materials and ultimately endow the Nb/SiO_2_-derived microporous membrane with sufficiently high H_2_/CO_2_ permselectivity. The exceptionally low permeance of CO_2_ is explained as a consequence of strong chemical interactions between CO_2_ and the materials of the membrane pore surface, presumably Nb-bound hydroxy groups. The results indicate that the H_2_/CO_2_ separation was based on sorption rather than on the differences in molecular sizes [26]. In other words, the existence of acid sites on the surface of membranes may play a key role in reducing CO_2_ permeance. The H_2_/CO_2_ and H_2_/N_2_ permselectivities of the Nb/SiO_2_ membrane with n_Nb_ = 0.08 obtained the maximum. Furthermore, with the further increase of n_Nb_, although there are still acid sites in the membrane materials, the formed Nb_2_O_5_ in the high-content Nb/SiO_2_ materials agglomerates to form a non-selective interfacial gap, which will lead to the densification of the membrane materials [13]. Thereby the average pore size will become smaller, resulting in the decrease of gas permeances and H_2_ permselectivities of the Nb/SiO_2_ membrane.

**Figure 14:**
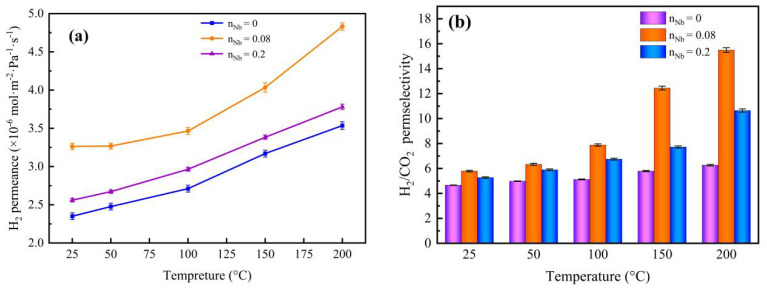
Effect of temperature on (**a**) H_2_ permeance and (**b**) H_2_/CO_2_ permselectivity of Nb/SiO_2_ membranes with different n_Nb_ at a pressure difference of 0.1 MPa.

Figure 14 displays the influence of temperature differences on the gas permeance of the Nb/SiO_2_ membrane with various n_Nb_ at a pressure difference of 0.1 MPa. As shown in Figure 14, the H_2_ permeance and H_2_/CO_2_ permselectivities in the Nb/SiO_2_ membranes with different n_Nb_ revealed an upward trend with increasing temperature when maintaining a constant pressure difference of 0.1 MPa, which indicated that the transport of H_2_ molecules through the membranes was activated.

**Figure 15:**
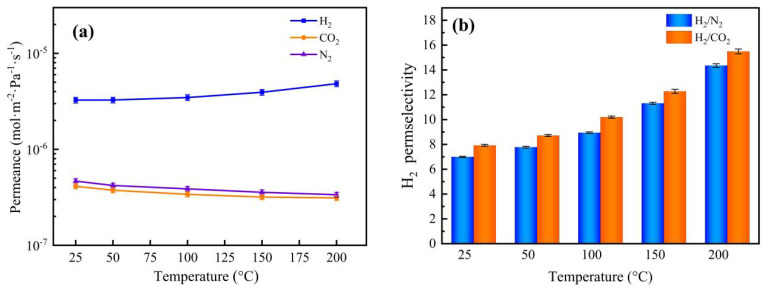
Effect of temperature on (**a**) gas permeance and (**b**) H_2_ permselectivity of Nb/SiO_2_ membrane with n_Nb_ = 0.08 at a pressure difference of 0.1 MPa.

The permeance and permselectivity of the Nb/SiO_2_ membrane with n_Nb_ = 0.08 at a pressure difference of 0.1 MPa and temperature change from 25 °C to 200 °C are manifested in Figure 15. It can be clearly seen that H_2_ permeance was significantly enhanced with increasing temperature, and the other permeances of CO_2_ and N_2_ were reduced slightly. The results were the same as the temperature dependency of permeance of several gases in the NS (Nb/SiO_2_) membrane from Boffa [27]. Thus, this gives rise to the permselectivity of H_2_/CO_2_ and H_2_/N_2_ elevating with increasing temperature. With increasing temperatures, the H_2_ permeance of the Nb/SiO_2_ membrane increased gradually, which shows that the permeation behavior of H_2_ in the membranes mainly follows an activation–diffusion mechanism. In the case of activated diffusion, molecules penetrate through the micropore while being subjected to a repulsive force from the pore walls, and the molecules with sufficient kinetic energy to overcome the repulsive force can penetrate the pores [16]. Conversely, the permeance of CO_2_ and N_2_ decreased slightly, similar to the trend of Knudsen diffusion, in which molecules collide with the pore walls more regularly than permeating molecules.

After the previous discussion, we believe that the permselectivity of Nb/SiO_2_ membranes has a sorption separation mechanism. High temperature is conducive to the permselectivity of H_2_, indicating that the permselectivity mechanism of H_2_ to other gases is mainly dominated by activation diffusion, which follows the Arrhenius equation.

(1) $$F=A_{0}\exp\left(\frac{-E_{a}}{\mathrm{RT}}\right)$$

In the formula, F means permeance, A_0_ means former factor, E_α_ means apparent activation energy, R means the ideal gas constant, T means the temperature, and Equation (1) can be described in another form:

(2) $$\mathrm{lnF}={\mathrm{lnA}}_{0}-\frac{E_{a}}{\mathrm{RT}}=A-\frac{E_{a}}{\mathrm{RT}}$$

**Figure 16:**
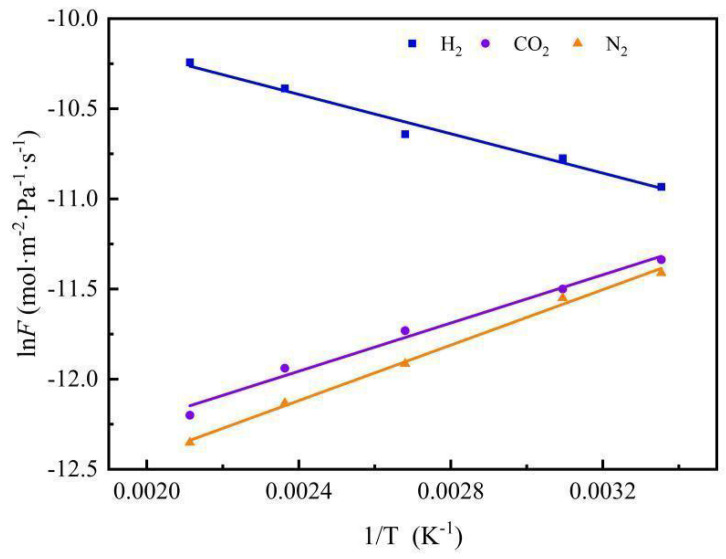
Arrhenius plots of gas (H_2_, CO_2_, and N_2_) permeances in Nb/SiO_2_ membrane with n_Nb_ = 0.08 at a pressure difference of 0.1 MPa.

The 1/T is used as the abscissa and lnF as the ordinate to draw the graph. It can be seen from the above formula that it is a straight line in theory. The apparent activation energy can be calculated from the slope of the straight line. Then the Arrhenius curves of the three gases are shown in Figure 16. Moreover, from the slope of the straight-line fitting in Figure 16, the apparent activation energy of the three gases can be obtained: E_m_ = E_a_ + Q_st_ [28]. The results are shown in Table 3.

From Table 4, Q_st_ means isosteric heat of adsorption, CO_2_ exhibits a greater adsorption heat, and the gas with the lowest adsorption heat is H_2_. The apparent activation energy (E_a_) can be positive or negative, depending on their relative magnitudes. A negative value of E_a_ is generally interpreted as being caused by strong sorption of the molecule on the pore surface. Such a negative value suggests a high enthalpy of sorption [26]. The mobility energy (E_m_) of gas molecules moving on the surface of the Nb/SiO_2_ membrane was E_m_ (CO_2_) > E_m_ (N_2_) > E_m_ (H_2_). The permselectivity of the Nb/SiO_2_ membrane towards H_2_/CO_2_ increased rapidly as a function of temperature. This was probably a result of the high activation energy of the mobility of hydrogen and the high heat of sorption of carbon dioxide. This result has never been reported for pure silica [29,30,31,32]. Thus, the strong heat of adsorption should be related to the presence of Nb ions in the microporous framework. Table 3 shows the E_a_ value of H_2_, H_2_ permeance, H_2_ permselectivities, and mean pore diameter of silica membranes by the sol–gel method from other researchers. From Table 3, it can be observed that it is difficult to improve the H_2_ permeance and permselectivity at the same time. In addition, a higher E_a_ always corresponds to a smaller mean pore diameter and lower H_2_ permeance. This means that the E_a_ value maybe have a link with the mean pore diameter and the interplay between the molecules of H_2_ and the pore walls of the membrane. Therefore, compared with other research groups, the Nb-doping in this work may be the reason for the lower E_a_ value of H_2_.

**Table 3:** E_a_ of H_2_, H_2_ permeances, H_2_ permselectivities, and mean pore diameter for various SiO_2_ membranes prepared by other researchers using the sol–gel process.

Membrane Type	Temperature and Pressure	E_a_ of H_2_ (kJ·mol^−1^)	H_2_ Permeance (mol·m^−2^·Pa^−1^·s^−1^)	H_2_ Permselectivities		Mean Pore Diameter (nm)	Ref.
				H_2_/CO_2_	H_2_/N_2_		
SiO_2_	200 °C, 2 bar	-	4.62 × 10^−7^	3.7	10.5	0.30–0.54	[33]
SiO_2_(400)	200 °C, 1 bar	8	17.4 × 10^−7^	7.5	64	0.38–0.55	[28]
SiO_2_(600)	200 °C, 2 bar	7.6	4.03 × 10^−7^	66	-	0.36–0.38	[28]
Pd/SiO_2_	200 °C, 0.3 MPa	-	7.26 × 10^−7^	4.3	14	0.57	[34]
Co/SiO_2_	200 °C, 0.2 MPa	1.98	1.97 × 10^−5^	10.48	13.08	2.34	[35]
Nb/SiO_2_ *	200 °C, 0.1 MPa	2.53	4.83 × 10^−6^	15.49	9.54	2.4549	

* In this work.

**Figure 17:**
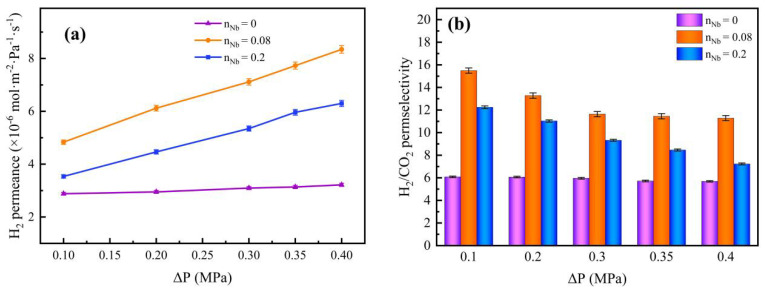
Effect of pressure difference on (**a**) H_2_ permeance and (**b**) H_2_/CO_2_ permselectivity of Nb/SiO_2_ membranes with different n_Nb_ at 200 °C.

The pressure dependence of various gas permeances and H_2_/CO_2_ permselectivities in the pure SiO_2_ membrane and Nb/SiO_2_ membrane with various n_Nb_ at 200 °C were further investigated in the pressure difference range from 0.10 MPa to 0.40 MPa, which is shown in Figure 17. It could be observed that H_2_ permeance with various n_Nb_ increased with the pressure-dependence increase. This is because the pressure difference elevated, and the gas force increased, resulting in an elevation in the gas concentration in the membrane, and then leading to higher H_2_ permeance. As seen in Figure 17b, the permselectivity of H_2_/CO_2_ changed slightly with increases in the pressure difference.

**Figure 18:**
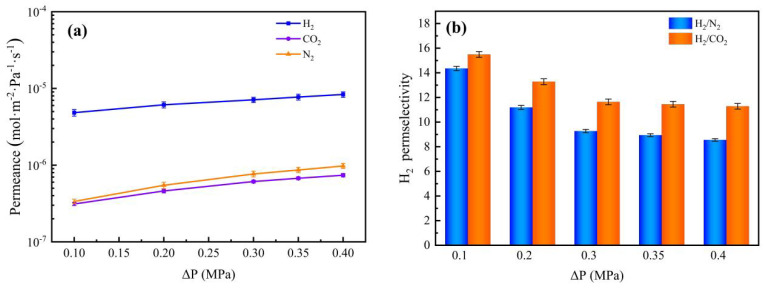
Effect of pressure difference on (**a**) gas permeances and (**b**) H_2_ permselectivities of Nb/SiO_2_ membrane at 200 °C.

Figure 18 demonstrates the effect of pressure differences on the permeance and permselectivity of different gases for the Nb/SiO_2_ membrane with n_Nb_ = 0.08 and a temperature at 200 °C. As seen in Figure 18a, as the pressure difference increased from 0.10 to 0.40 MPa, all of the gas permeances increased. The reason is that the increases in intake pressure resulted in an elevation of the gas concentration, thus yielding a higher permeance. Furthermore, the relationship between permselectivity and pressure differences is shown in Figure 18b. For example, with the pressure increasing from 0.10 MPa to 0.40 MPa, the permeance of N_2_ and CO_2_ slightly increased. This was due to the small influence of pressure on Knudsen diffusion, as previously reported in the literature [30]. Hence, no matter how high the pressure was, the change in permselectivity would not increase significantly.

Traditional SiO_2_ membranes have poor hydrothermal stability due to a large amount of Si-OH groups on their surfaces, which easily absorb water vapor in the air. In the Nb/SiO_2_ membrane, not only the Nb-doping but also the introduced hydrophobic groups can improve the vapor stability, which can reduce the hydroxyl groups on the pore surface and enhance the hydrophobicity. In order to test the hydrothermal stability of membranes, the Nb/SiO_2_ membranes with n_Nb_ = 0 and 0.08 were chosen to investigate the gas permeance before and after steam treatment.

**Figure 19:**
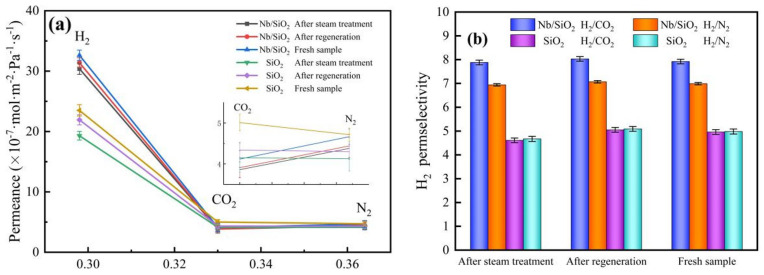
(**a**) Gas permeance and H_2_ permselectivities and (**b**) H_2_ permselectivities of Nb/SiO_2_ membranes with n_Nb_ = 0 and 0.08 at a pressure difference of 0.1 MPa before and after steam treatment and regeneration.

Figure 19 shows the permeances of various gases (H_2_, CO_2_, and N_2_) and H_2_ permselectivities of Nb/SiO_2_ membranes with n_Nb_ = 0 and 0.08 at 25 °C and a pressure difference of 0.1 MPa before and after steam treatment and regeneration. As shown in Figure 19, compared with the fresh membranes, the H_2_ permeances of SiO_2_ and Nb/SiO_2_ membranes after steam treatment decreased by 17.90% and 6.68%, respectively, while their H_2_/CO_2_ permselectivities decreased by 3.2% and increased by 1.6%, respectively. After regeneration by calcination at 350 °C, the gas permeances and the permselectivities of H_2_/CO_2_ and H_2_/N_2_ for the two membranes showed an upward trend. Compared with the fresh membranes, the H_2_ permeances of SiO_2_ and Nb/SiO_2_ membranes after regeneration decreased by 10.95% and 3.21%, respectively, while their H_2_/CO_2_ permselectivities increased by 2.8% and 2.1%, respectively. The above results indicate that niobium doping improves the hydrothermal stability of SiO_2_ membranes.

## 4. Conclusions

To sum up, using the sol-gel technique, Nb/SiO_2_ materials and membranes with various n_Nb_ were successfully synthesized. Their microstructures and gas permeances were investigated. The results showed that the niobium element existed in the formation of the Nb-O groups in the Nb/SiO_2_ materials. As the niobium doping content and the calcining temperature were high enough, the Nb_2_O_5_ crystals could be formed in the SiO_2_ frameworks. With the increase of n_Nb_, the formed particle sizes increased, and the mean pore size, BET surface area, and total pore volume also increased until n_Nb_ = 0.08, and then they began to decrease. The doping of Nb could enhance the H_2_/CO_2_ and H_2_/N_2_ permselectivities of the SiO_2_ membrane. When n_Nb_ was equal to 0.08, the Nb/SiO_2_ membrane achieved a maximal H_2_ permeance of 4.83 × 10^−6^ mol·m^−2^·Pa^−1^·s^−1^ and H_2_/CO_2_ permselectivity of 15.49 at 200 °C and 0.1MPa, which increased by 36.7% and 155.47%, respectively, compared with that of the pure SiO_2_ membrane. Compared with the fresh membranes, the H_2_ permeances of SiO_2_ and Nb/SiO_2_ membranes after steam treatment decreased by 17.90% and 6.68%, respectively, while their H_2_/CO_2_ permselectivities decreased by 3.2% and increased by 1.6%, respectively. After regeneration, the gas permeances and the permselectivities of H_2_/CO_2_ and H_2_/N_2_ for the two membranes showed an upward trend. Compared with the fresh membrane, the H_2_ permeances of SiO_2_ and Nb/SiO_2_ membranes after regeneration decreased by 10.95% and 3.21%, respectively, while their H_2_/CO_2_ permselectivities increased by 2.8% and 2.1%, respectively. Niobium doping improved the hydrothermal stability of the SiO_2_ membrane. The Nb/SiO_2_ membranes also exhibited great thermal reproducibility.

## Figures and Tables

**Table 1:** The influence of n_Nb_ on the pH value, density, and solid content of Nb/SiO_2_ sol.

n_Nb_	pH	Density/g·cm^−3^	Solid Content/%
0	3.41 ± 0.04	0.8418 ± 0.0007	22.31 ± 0.04
0.08	2.93 ± 0.03	0.8529 ± 0.0006	22.58 ± 0.05
0.2	2.64 ± 0.02	0.8710 ± 0.0008	22.80 ± 0.06
1	1.02 ± 0.02	0.9130 ± 0.0006	24.46 ± 0.07

**Table 2:** Pore structure parameters of Nb/SiO_2_ materials with various n_Nb_ calcined at 400 °C.

n_Nb_	BET/ m^2^·g^−1^	V_t_/ cm^3^·g^−1^	V_mic_/ cm^3^·g^−1^	Mean Pore Width/nm
0	386.4545	0.1716	0.1115	1.8759
0.08	778.7121	0.4901	0.0867	2.4549
0.2	535.4072	0.4632	0.0748	2.2591
1	86.1599	0.0762	0.0266	1.2176

**Table 4:** E_a_, Q_st_, and E_m_ values of gases (H_2_, CO_2_, and N_2_) calculated from the Arrhenius formula for the Nb/SiO_2_ membrane with n_Nb_ = 0.08 at a pressure difference of 0.1 MPa.

Gases	E_a_/ kJ·mol^−1^	Q_st_/ kJ·mol^−1^ [28]	E_m_/ kJ·mol^−1^
H_2_	2.53	6	8.53
CO_2_	−4.28	24	19.72
N_2_	−4.07	18	13.93

## Data Availability

Not applicable.
